# Multiple Enhanced Sparse Representation via IACMDSR Model for Bearing Compound Fault Diagnosis

**DOI:** 10.3390/s22176330

**Published:** 2022-08-23

**Authors:** Long Zhang, Lijuan Zhao, Chaobing Wang, Qian Xiao, Haoyang Liu, Hao Zhang, Yanqing Hu

**Affiliations:** School of Mechatronics & Vehicle Engineering, East China Jiaotong University, Nanchang 330013, China

**Keywords:** compound fault diagnosis, feature extraction, IACMDSR, vibration-based analysis

## Abstract

For the sake of addressing the issue of extracting multiple features embedded in a noise-heavy vibration signal for bearing compound fault diagnosis, a novel model based on improved adaptive chirp mode decomposition (IACMD) and sparse representation, namely IACMDSR, is developed in this paper. Firstly, the IACMD is employed to simultaneously separate the distinct fault types and extract multiple resonance frequencies induced by them. Next, an adaptive bilateral wavelet hyper-dictionary that digs deeper into the periodicity and waveform characteristics exhibited by the real fault impulse response is constructed to identify and reconstruct each type of fault-induced feature with the help of the orthogonal matching pursuit (OMP) algorithm. Finally, the fault characteristic frequency can be detected via an envelope demodulation analysis of the reconstructed signal. A simulation and two sets of experimental results confirm that the developed IACMDSR model is a powerful and versatile tool and consistently outperforms the leading MCKDSR and MCKDMWF models. Furthermore, the developed model has satisfactory capability in practical applications because the IACMD has no requirement for the input number of the signal components and the adaptive bilateral wavelet is powerfully matched to the real fault-induced impulse response.

## 1. Introduction

Rolling bearing is a widely distributed component in a plethora of rotating machinery. As a key component, its availability and reliability are critical to ensure the effective and safe operation of the entire mechanical system [1,2]. Bearing failure can cause not only unscheduled downtime and economic loss but also serious injury or death. In practical engineering applications, due to the close-coupling of mechanical parts in a rotating machine, a single fault may eventually lead to abnormal behavior of other units. Therefore, the bearing is typically accompanied by a compound fault in which multiple faults occur at the same time [3]. Even worse, the bearing usually operates in harsh working conditions, leading fault-induced impulse responses that are easily disturbed by external interference and ambient noise. Thus, the multi-feature extraction of bearing compound faults has received widespread attention.

Vibration-based analysis has become one of the most active and valid means of fault feature extraction for bearings, because a fault vibration signal directly transmits dynamic information regarding not only the fault condition but also the fault type [4]. Many advanced vibration-based procedures have yielded fruitful results in fault feature extraction, such as wavelet transform [5], recurrence analysis [6,7], maximum correlated kurtosis deconvolution (MCKD) [8], empirical mode representation (EMD) [9], tunable-Q factor wavelet transform (TQWT) [10], deep learning, and sparse representation [11]. More specifically, Liu et al. [12] combined EMD with sparse representation to identify the gear local fault feature. Firstly, the EMD is employed to obtain the prominent impact from the resulted of the intrinsic mode function (IMF) based on the kurtosis index. Next, the correlation filtering algorithm (CFA) was applied to obtain the dictionary parameters from the optimal IMF. Finally, they successfully extracted the gear local fault features. Similarly, Wang et al. [13] put forward a method for detecting bearing early weak faults by combining EEMD and TQWT. Moreover, Li et al. [14] utilized the improved TQWT method to select an atom to build a self-adaptive dictionary to sparsely represent the fault features and demonstrated that the method is appropriate for extracting bearing fault features. These methods show outstanding performance in single-fault feature extraction but are less satisfactory in compound fault diagnosis. Research has been conducted on improving these methods to meet the demands of multiple feature extraction. For example, Deng et al. [15] designed a methodology for bearing compound fault diagnosis by employing optimized MCKD and sparse representation. Gao et al. [16] applied the MCKD with a convolutional neural network (CNN) to detect bearing compound faults. The MCKD is used for pre-filtering, and then the denoised signal is input to the CNN to realize fault identification and classification. Hong et al. [17] integrated the improved MCKD with multi-wavelet transform to diagnose compound faults in rotating machinery. Yan et al. [18] applied the optimal variational mode representation (VMD) as the pre-filter to select two IMFs, then a 1.5-dimension envelope spectrum was employed to detect the characteristic features of the rotating machinery compound faults. Nevertheless, the above two-step strategy used in the compound fault diagnosis method has some drawbacks:

(1) The pre-processing and post-processing are carried out separately, making them computationally expensive.
(2) The critical parameters of the procedures are required to be specified by humans, demanding a certain degree of expertise and thus with some randomness.
(3) The de-noising capability of these combination methods is inadequate and thus a considerable level of residual noise remains in the extracted fault-induced impulse responses.

In the case of difficult-to-separate complicated signals, the ACMD [19] employs a greedy algorithm to accurately estimate the instantaneous amplitude (IA) and instantaneous frequency (IF) for each signal component individually, and thus it can simultaneously extract multiple resonance frequencies and not require the number of signal components to be entered in advance. However, the different signal components separated by ACMD are still disturbed by noise. The sparse representation performs pre-eminently in bearing fault feature extraction. It is worth pointing out that building a dictionary that matches the fault impulse of interest and determining the coefficients with an appropriate algorithm are essential for sparse representation. Li et al. [20] developed a period-assisted wavelet dictionary and combined it with the OMP algorithm to represent the fault-induced impulse responses of rolling bearings. Kong et al. [21] put forward an enhanced intelligent recognition method based on sparse representation for planet-bearing fault diagnosis. Yang et al. [22] introduced a multi-featured sparse representation method for gearbox fault diagnosis on the basis of a double-dictionary and split augmented Lagrangian shrinkage algorithm (SALSA).

Based on the foregoing analysis, an IACMD conjunct with sparse representation known as the IACMDSR model is designed with the aim to diagnose bearing compound fault. Firstly, the IACMD is applied to extract the multiple resonance frequencies induced by distinct fault types, as well as to achieve the initial separation of the various fault-induced compound signals. Furthermore, a sparse representation is employed to reconstruct the transient impulse response of various signal components. The main contributions of IACMDSR are described as follows:

(1) The IACMD is not only used to separate compound faults; its extracted fault-induced resonance frequencies can be embedded in a dictionary for sparse decomposition, saving computational cost and ensuring the accuracy of the IACMDSR model.
(2) The IACMD also enables the separation of compound signals without requiring the number of signal components a priori, making it suitable for engineering applications that do not know the number of signal components in advance.
(3) In contrast to popular sparse decomposition dictionaries that adopt the wavelet atoms with a single damping ratio (for example, the Laplace wavelet [23] and the Morlet wavelet [24]) as the basis functions for atoms, this paper employs period-assisted bilateral wavelets with a double damping ratio to form the atoms, which can better match the period-occurred impulse response of bilateral asymmetric attenuation caused by actual faults and thus improve the accuracy of sparse representation.

The remainder of this paper is arranged as follows: Section 2 briefly introduces the IACMD and the design of the bilateral adaptive wavelet hyper-dictionary. The detailed introduction of the IACMDSR model for multiple feature extraction is given in Section 3. In Section 4, the simulation compound signal analysis is given to demonstrate the effectiveness of the IACMDSR model. Two sets of experimental compound signals are further analyzed in Section 5 to validate the outperformance of the IACMDSR model, and two leading contrastive studies are performed to show the superiority of the IACMDSR model. Finally, the conclusion is presented in Section 6.

## 2. The Framework of IACMDSR Model

### 2.1. The IACMD Algorithm

A non-stationary vibration signal s(t) composed of *M* signal components can be modeled as [19]

(1) $$s\left(t\right)=\sum_{i=1}^{M}s_{i}\left(t\right)=\sum_{i=1}^{M}A_{i}\left(t\right)\cos(2\pi \int_{0}^{t}f_{i}\left(\tau \right)d\tau +\theta_{i})$$

where $A_{i}$, $f_{i}$, and $\theta_{i}$ represent the IA, the IF, and the initial phase of the *i*-th signal component $s_{i}\left(t\right)$, respectively.

With the help of the signal demodulation technique, Equation (1) can be rewritten as

(2) $$s\left(t\right)=\sum_{i=1}^{M}a_{i}\left(t\right)\cos\left(2\pi \int_{0}^{t}\tilde{f_{i}}\left(\tau \right)d\tau \right)+b_{i}\left(t\right)\sin\left(2\pi \int_{0}^{t}\tilde{f_{i}}\left(\tau \right)d\tau \right)$$

where

(3) $$\{\begin{array}{c} a_{i}\left(t\right)=A_{i}\left(t\right)\cos(2\pi \int_{0}^{t}\left(f_{i}\left(\tau \right)-\tilde{f_{i}}\left(\tau \right)\right)d\tau +\theta_{i})\\ b_{i}\left(t\right)=-A_{i}\left(t\right)\sin(2\pi \int_{0}^{t}\left(f_{i}\left(\tau \right)-\tilde{f_{i}}\left(\tau \right)\right)d\tau +\theta_{i}) \end{array}$$

where $\tilde{f_{i}}\left(\tau \right)$ denotes the frequency function of the two demodulation operators $\cos(2\pi \int_{0}^{t}\tilde{f_{i}}\left(\tau \right)d\tau )$ and $\sin(2\pi \int_{0}^{t}\tilde{f_{i}}\left(\tau \right)d\tau )$; $a_{i}\left(t\right)$ and $b_{i}\left(t\right)$ are the demodulated signals which are used to recover the IA of the signal components as

(4) $$A_{i}\left(t\right)=\sqrt{a_{i}{}^{2}+b_{i}{}^{2}}$$

Motivated by the VMD algorithm and matching pursuit [25], the ACMD employs a greedy approach to separate the signal components one by one. For the *i*-th signal component, the representation problem can be modeled as

(5) {‖ai″(t)‖22+‖bi″(t)‖22+α‖s(t)−si(t)‖22}ai(t),bi(t),fi˜(t)mins.t. si(t)=ai(t)cos(2π∫0tfi˜(τ)dτ)+bi(t)sin(2π∫0tfi˜(τ)dτ)

where s(t) denotes the input signal; $s_{i}\left(t\right)$ is the objective component to be extracted. $\|s\left(t\right)-s_{i}\left(t\right){\|}_{2}^{2}$ represents the residue signal after the objective component has been removed. α denotes the weighting coefficient. In fact, the ACMD is essentially an adaptive bandpass filter, and the corresponding signal components can be estimated as

(6) $$s_{i}^{k}=G_{i}^{k}u_{i}^{k}$$

(7) $$R_{i}\left(t\right)=s\left(t\right)-\tilde{s_{i}}\left(t\right)$$

where $R_{i}\left(t\right)$ is the residue signal component and $\tilde{s_{i}}\left(t\right)$ is the *i*-th modal component. According to Equation (3), the frequency increment can be characterized as

(8) $$\Delta \tilde{f_{i}}\left(t\right)=-\frac{1}{2\pi }\frac{d}{dt}\left(\arctan\left(\frac{b_{i}^{k}\left(t\right)}{a_{i}^{k}\left(t\right)}\right)\right)=\frac{b_{i}^{k}\left(t\right)\times {\left(a_{i}^{k}\left(t\right)\right)}^{\prime }-a_{i}^{k}\left(t\right)\times {\left(b_{i}^{k}\left(t\right)\right)}^{\prime }}{2\pi ({\left(a_{i}^{k}\left(t\right)\right)}^{2}+\left(b_{i}^{k}\left(t\right){)}^{2}\right)}$$

Note that the Equation (8) is expected to be sufficiently smooth. Thus, a low-pass filter is utilized to pre-process the frequency increment in minimizing the interference components. Finally, the IF is shown as

(9) $$f_{i}^{k+1}=f_{i}^{k}+{\left(\frac{1}{\beta }\Omega^{T}\Omega +I\right)}^{-1}\Delta {\tilde{f}}_{i}^{k}$$

where $f_{i}^{k}={\left[{\tilde{f}}_{i}^{k}\left(t_{0}\right),\;L,\;{\tilde{f}}_{i}^{k}\left(t_{N-1}\right)\right]}^{T}$, $\Delta {\tilde{f}}_{i}^{k}={\left[\Delta {\tilde{f}}_{i}^{k},L,\Delta {\tilde{f}}_{i}^{k}\left(t_{N-1}\right)\right]}^{T}$, I is an identity matrix, Ω is the second-order difference matrix, and ${\left(1/\beta \Omega^{T}\Omega +I\right)}^{-1}$ can be seen as the low-pass filter. As can be seen from Equation (9), the ACMD generates multiple IFs in the process of decomposing the compound signal. In order to provide an overall measurement of the resonance frequency generated by the fault, we made improvements to the ACMD. The original multiple IFs are averaged to represent the resonance frequencies derived from faults, thus the IF can be described as:

(10) $$f=\frac{1}{i}\left[f_{i}^{1}+f_{i}^{2}+\cdots +f_{i}^{n}\right]=\frac{1}{i}\sum_{n=1}^{i}f_{i}$$

The strengths of the IACMD in extracting the fault-induced resonance frequency is demonstrated in the simulation compound signal in Section 4.

### 2.2. The Improved Sparse Optimization Model

The sparse representation model for a single component fault objective signal $y\in R^{N}$ with a noise component can be expressed as

(11) y=Dx+noise

where *D* $\left(d_{1},d_{2},\ldots ,d_{M}\right)$ (M>N) is a sparse representation dictionary and x is the sparsity coefficient. The aim of signal sparse representation has always been to choose as few atoms ($d_{i})$ as possible in an overcomplete dictionary *D* to represent all or most of the fault impulse responses. It can be seen from Equation (11) that the sparse representation consists of two main parts: ① dictionary construction, the higher the similarity and matching of the dictionary atom $d_{i}$ to the fault impulse response, the better the sparse representation and ② the determination of the sparse coefficients, the speed of the sparse representation is mainly affected by the algorithm [26].

#### Bilateral Adaptive Wavelet Hyper-Dictionary

**Figure 1:**
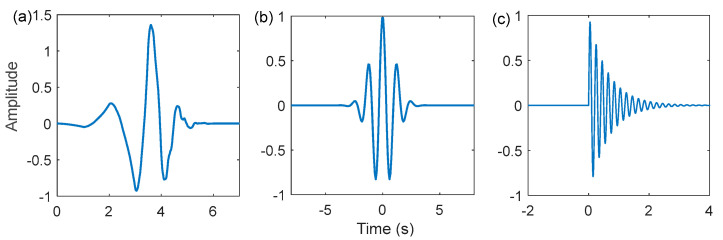
A few typical wavelets: (**a**) db4 wavelet; (**b**) Morlet wavelet; (**c**) Laplace wavelet.

**Figure 2:**
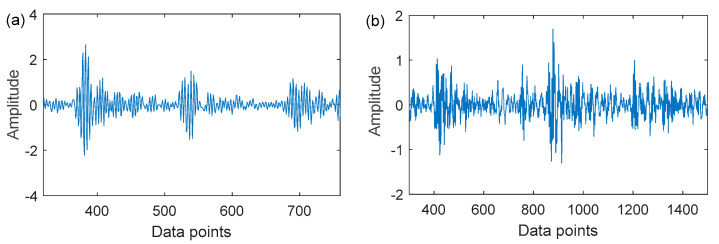
Bearing fault signals: (**a**) outer ring fault; (**b**) inner ring fault.

**Figure 3:**
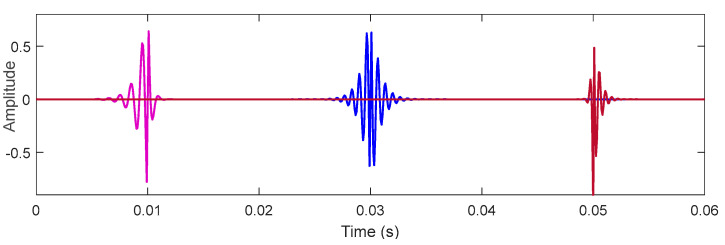
Waveforms of the bilateral wavelets with different parameter combinations.

Wavelet dictionaries have been the subject of extensive research over the past few years due to the flexibility and versatility of wavelet waveforms [27,28]. Among them, the Laplace wavelet dictionary, and the Morlet wavelet dictionary have made remarkable achievements in the sparse representation of fault features. Figure 1 illustrates the time domain waveforms of a few typical wavelets. Nevertheless, the wavelets used in the above dictionary atoms are all single-damped wavelets showing the waveform of unilateral decay or bilateral symmetrical attenuation. Moreover, the impulse response of a real vibration signal is distorted to some extent by the transmission path and noise, resulting in a bilateral asymmetric attenuation shape. In Figure 2, the real signals of the inner and outer rings of the failed bearing of the Case Western Reserve University also confirm this phenomenon. Therefore, to improve the matching of the dictionary atoms to the fault impulse response, period-assisted bilateral wavelets are employed to design the hyper-dictionary. The expression of the bilateral adaptive wavelet is as follows:

(12) $$\begin{array}{c} g_{imp}\left(\omega ,\xi ,\zeta ,\tau ,t\right)=\left\{\begin{array}{c} K_{imp}\left(t-\tau ,0\right)\cdot e^{\frac{-\xi }{\sqrt{1-\xi^{2}}}\omega \left(t-\tau \right)}\cos\left(\omega \left(t-\tau \right)\right)-K_{imp\;}\left(\tau -t,0\right)\cdot e^{\frac{\zeta }{\sqrt{1-\zeta^{2}}}}\cos\left(\omega \left(t-\tau \right)\right),t\in \left[\tau -W_{s},\tau +W_{s}\right]\\ 0,\;else \end{array}\right.\\ K_{imp\;}\left(t,0\right)=\left\{\begin{array}{cc} 1, & \;\;\;t>0\\ 0, & \;\;\;else \end{array}\right. \end{array}$$

where ω=2πf denotes the oscillation frequency, which is represented by f in the following equation. Additionally, ξ and ζ denotes the damping ratios that decide oscillation attenuation on the left and right sides of the wavelet, respectively. τ is the time-shift, which decides the position of the wavelet on the *X*-axis. Figure 3 depicts three bilateral wavelet waveforms with different parameter combinations, where the parameters are $g_{imp}\left(1000,\;0.5,\;0.2,\;0.01\right)$, $g_{imp}\left(1500,\;0.15,\;0.15,\;0.03\right)$, and $g_{imp}\left(1800,\;0.2,\;0.5,\;0.05\right)$, respectively.

The resonance frequency for each fault type obtained by the IACMD is adopted as the oscillation frequency $f_{i}$ of the corresponding bilateral wavelet; then, the CFA [29] is integrated with the WOA to adaptively locate the optimal damping ratios $\xi_{i}$ and $\zeta_{i}$ of the wavelet that is most similar to the fault impulse response. First, the parameters of the WOA are initialized, the population size is set up as 20, and the maximum number of iterations is set up as 60. Considering that the viscous damping ratio in steel structures is generally less than 0.2, the ranges of ξ and ζ are both (0, 0.2). Finally, identifying the optimal parameters ξ and ζ by finding the maximum correlation coefficient $C_{\gamma }$ (the fitness function of the WOA) between the atom $d_{i}$ and the constructed bilateral wavelet $g_{imp}$. The mathematical expression of $C_{\gamma }$ is shown in Equation (13). It is worth pointing out that, in the literature [27,30], the dictionary atom is usually made up of a single optimal wavelet. Taking into account the periodic characteristic of the fault impulse response, period-assisted bilateral wavelets are employed as the atoms $d_{i}$. The length of a single wavelet $L_{s}$: $L_{s}=f_{s}/f_{i}$ (where the $f_{s}$ is the sampling frequency, $f_{i}$ is the fault frequency), and the period number *N* is set as 4 [20]. Finally, by changing the time-shift τ of the wavelet atoms corresponding to different fault components, a bilateral adaptive wavelet compound dictionary *D* is designed, which can be expressed as Equation (14)

(13) $$C_{\gamma }=\frac{\left|d_{i},\;g_{imp}\right|}{\|d_{i}{\|}_{2}\|g_{imp}{\|}_{2}}$$

(14) $$D=\{\begin{array}{c} \begin{array}{c} D_{1}=\left\{g_{imp}(f_{1},\xi_{1},\zeta_{1},\tau \right\}\\ D_{2}=\left\{g_{imp}(f_{2},\xi_{2},\zeta_{2},\tau \right\} \end{array}\\ \begin{array}{c} \vdots \\ D_{i}=\left\{g_{imp}(f_{i},\xi_{i},\zeta_{i},\tau \right\} \end{array} \end{array}$$

## 3. Fault Diagnosis Procedure Based on IACMDSR Model

**Figure 4:**
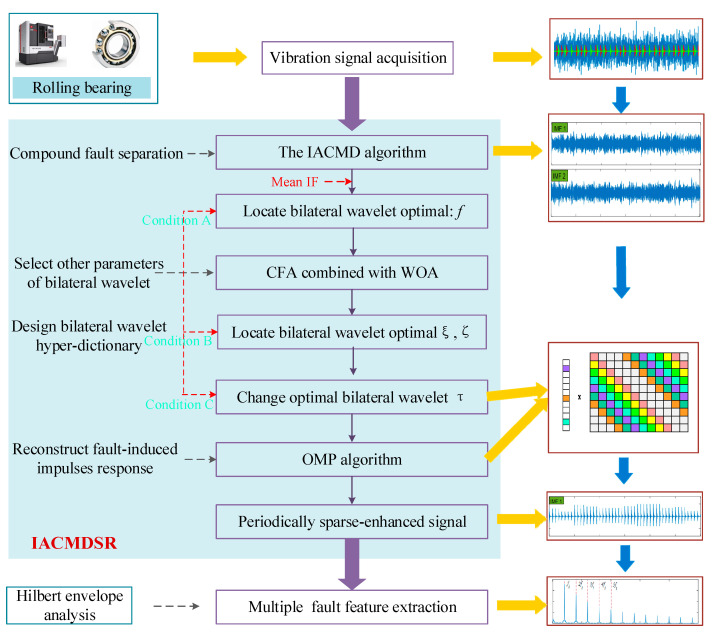
The procedure of IACMDSR for extracting multiple bearing fault feature.

With the analysis mentioned above, the novel IACMDSR model is proposed for multiple fault feature extraction based on ACMD and sparse representation, which mainly includes four procedures. The flowchart is shown in Figure 4.

(1) The first step is to use the IACMD to adaptively separate the compound fault signal y into different fault signal components based on the amplitude of the signal spectrum. The IACMD not only separates the different fault signal components but also extracts the multiple resonance frequencies generated by each fault type.
(2) Second is the design of bilateral adaptive wavelet hyper-dictionary *D* based on the method mentioned in Section 2.2.
(3) Third is the use of the OMP algorithm and hyper-dictionary to reconstruct each of the signal components decomposed by the IACMD. In which, in order to improve computational efficiency, each signal component is divided into $y_{i}$ segments, and the length of each segment is $N\times L_{s}$. Then, the OMP algorithm is employed to obtain a sparse coefficient matrix $\alpha =\left(\alpha_{1},\alpha_{2},\ldots ,\;\alpha_{3}\right)$. The objective function is shown as follows:
  (15) αi=‖αargminDα−yi‖22+μ‖a‖0
  Then, the reconstructed signal $\hat{x}$ can be expressed as:
  (16) x^=λxargmin‖x−y‖22+∑i‖Dαi−yi‖22
(4) The Hilbert transform and square operation is applied to the reconstructed signal $\hat{x}$ to obtain the squared envelope spectrum. Ultimately, the fault characteristic frequencies can be identified from the envelope spectrum.

## 4. Simulation Signals and Analysis

A bearing simulation signal is constructed and carried out to illustrate the effectiveness of the IACMDSR model on compound fault feature extraction. The bearing fault signal is made up of two periodic impulse signals and a noise signal. The description of the simulation signal can be described as

(17) $$\{\begin{array}{c} \begin{array}{c} x\left(t\right)=x_{1}\left(t\right)+x_{2}\left(t\right)+n\left(t\right)\\ x_{1}\left(t\right)=\overset{M}{\underset{i=1}{\sum }}A_{m}h_{1}(t-iT_{r1})\\ x_{2}\left(t\right)=\overset{M}{\underset{i=1}{\sum }}A_{m}h_{2}(t-iT_{r2}) \end{array}\\ h\left(t\right)=e^{-\left(\xi_{i}/\sqrt{1-\xi_{i}^{2}}\right)2\pi f_{i}} \end{array}$$

where $x_{1}\left(t\right)$ and $x_{2}\left(t\right)$ denotes the bearing inner and outer ring fault signal, respectively. n(t) is the white noise with a standard deviation of 1.2 and $h\left(t-iT_{r}\right)$ represents the periodic impulse responses. The amplitude parameter $A_{m}$ is 1. The attenuation coefficient $\xi_{1}$ is 600, $\xi_{2}$ is 800; the resonance frequency is $f_{1}$ = 2000 Hz and $f_{2}=3500$ Hz; and the fault characteristic frequency is $f_{1}=1/T_{r1}$ = 80 Hz and $f_{2}=1/T_{r2}$ = 60 Hz. Furthermore, the sampling frequency is $f_{s}=12\;\mathrm{kHz}$.

**Figure 5:**
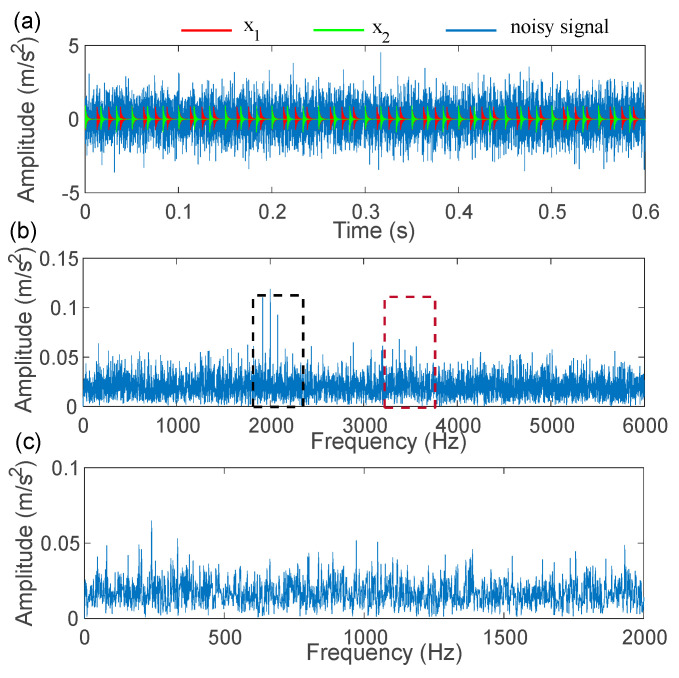
Compound fault simulation signals: (**a**) compound signal; (**b**) spectrum; (**c**) envelope spectrum.

**Figure 6:**
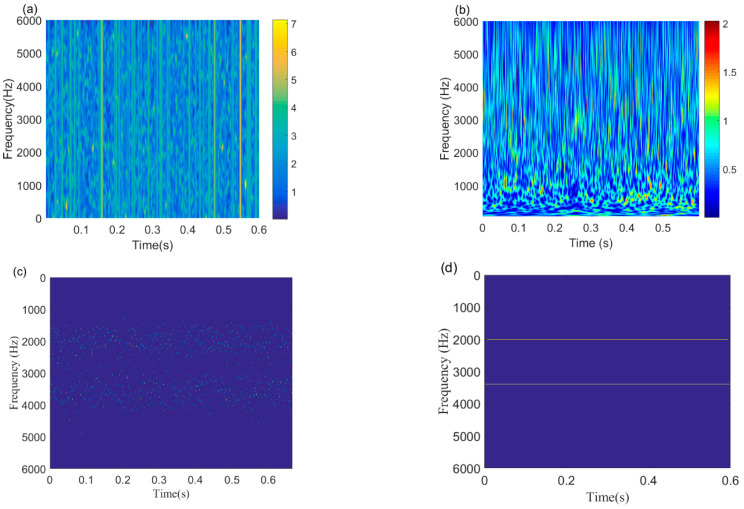
Time-frequency feature extraction results of commonly used methods: (**a**) STFT; (**b**) CWT; (**c**) ACMD; (**d**) IACMD.

The time-domain waveforms, the spectrum, and the envelope spectrum of the simulation compound signals are depicted in Figure 5. As illustrated in Figure 5a, the fault impulse responses of different components are buried in the noise. In the spectrum presented in Figure 5b, the information of the fault modulation band can be detected near the resonance frequencies of 2000 Hz and 3500 Hz. However, in the envelope spectrum that is depicted in Figure 5c, there is no fault frequency or its harmonics. Subsequently, the IACMDSR model is employed for the simulation compound signals. Firstly, the IACMD is employed to isolate the various signal components. In the time-frequency graph shown in Figure 6d, the resonance frequencies $f_{1}$ = 2000 Hz and $f_{2}=3500$ Hz of the simulation compound signal are clearly extracted by the IACMD.

For composition, some leading methods are also employed to extract the resonance frequency. Figure 6a,b shows the resonance frequency results of the STFT and CWT methods, respectively. Considering that the Morlet wavelet waveform has a shape similar to that of a bearing fault-induced impulse response, the Morlet wavelet is employed as the mother wavelet of the CWT. As observed in the time-frequency graphs, the fault resonance frequencies are not reflected by STFT and CWT. Since the frequency of the noise simulation signal varies considerably, the STFT window is fixed and cannot be adaptively adjusted based on the variations of the signal. Therefore, the accuracy of time-frequency positioning is limited. The window of the CWT is a variable window determined by a scale factor. However, an appropriate mother wavelet is difficult to choose and is susceptible to noise. The original ACMD extraction result is shown in Figure 6c. The ACMD employs a greedy algorithm to capture each signal component individually. Hence, we obtain a high-resolution adaptive time-frequency spectrum that clearly represents the fault-induced characteristic frequency by using the estimated IA and the IF. However, we can see that the IFs of each signal component obtained by the ACMD tends to fluctuate significantly around the fault resonance frequency. Thus, in the IACMD, we take the average of multiple IFs as the fault resonance frequency, enabling the better visualization of the result (Figure 6d). From the above analyses, the benefit of the IACMD is demonstrated.

**Figure 7:**
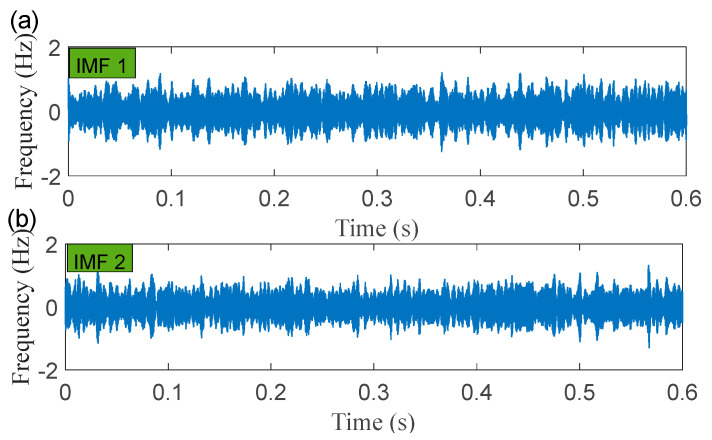
The processing results of the IACMD: (**a**) time-domain waveform of IMF 1; (**b**) time-domain waveform of IMF 2.

**Figure 8:**
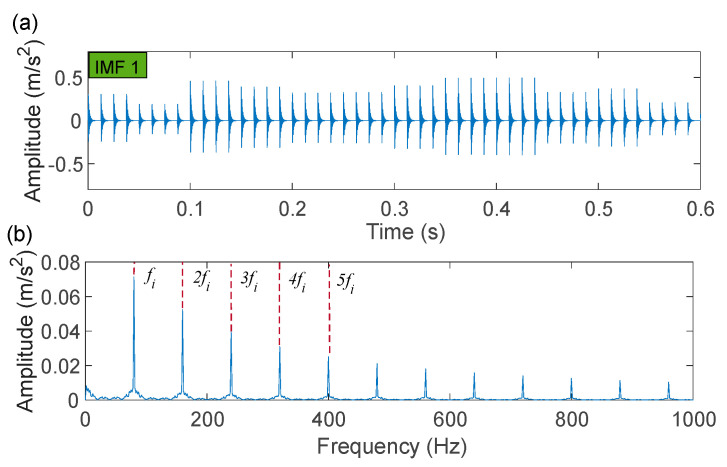
The IACMDSR model results of IMF1 (inner ring fault): (**a**) time-domain waveform; (**b**) envelope spectrum.

**Figure 9:**
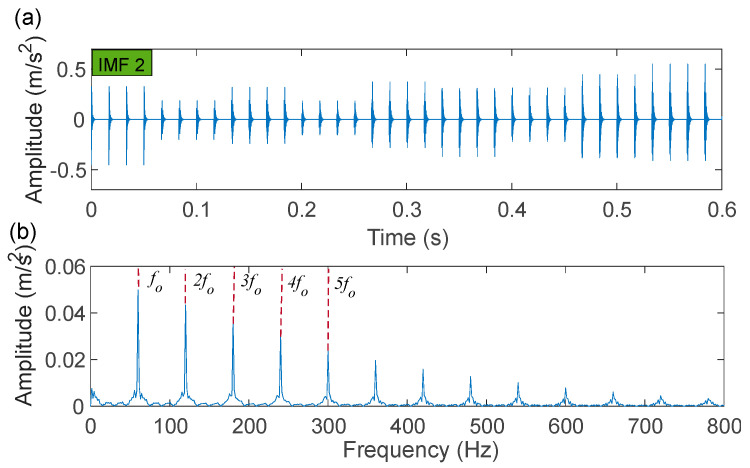
The IACMDSR model results of IMF2 (outer ring fault): (**a**) time-domain waveform; (**b**) envelope spectrum.

**Figure 10:**
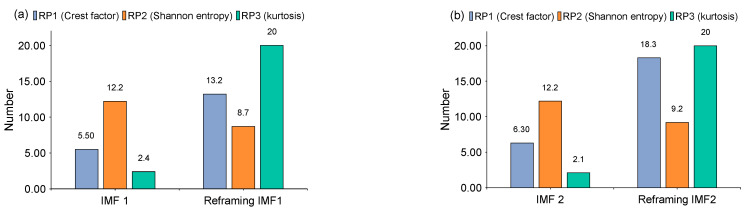
The index results of ACMD decomposition (Figure 7) and IACMDSR reconstruction (Figure 8 and Figure 9) (**a**) IMF 1; (**b**) IMF 2.

The time-domain waveforms of the corresponding in-band signal components are displayed in Figure 7a,b. We can discover from the time-domain waveforms that the fault signal components are disorderly. Next, a hyper-dictionary is designed to provide a sparse representation of the different signal components. The first step in building the hyper-dictionary is identifying the optimal parameters of the bilateral wavelet for the dictionary atom. The resonance frequencies $f_{1}$ = 2000 Hz, $f_{2}=3500$ Hz obtained by IACMD are utilized as the oscillation frequency of the corresponding wavelet. The damping ratio parameters searched by the CFA are as follows: $\xi_{1}=0.0708$ and $\zeta_{1}\;=\;0.0571$; $\xi_{2}=0.0588$ and $\zeta_{2}=0.0168$. The time-domain waveforms of the inner ring fault signal (IMF1) and the outer ring fault signal (IMF2) after the IACMDSR model are displayed in Figure 8a and Figure 9a, respectively. Noise interference is well inhibited, and almost all the periodic impulse responses can be noticed. In the envelope spectrum shown in Figure 8b and Figure 9b, the fault frequency and its multiplier are both clearly visible. To show the importance of the sparse decomposition process in the IACMDSR model, as shown in Figure 10, the crest factor, Shannon entropy, and Kurtosis are adopted to evaluate the quality of the signal components derived from IACMD decomposition and IACMDSR reconstruction. In Figure 10, we can see that the signal quality has dramatically improved after the reconstruction of the IACMDSR model. Meanwhile, the efficiency of the IACMDSR in distinguishing compound faults is well confirmed.

## 5. Application Verification

To demonstrate the effectiveness and superiority of IACMDSR in separating the compound fault of the bearings, the run-to-failure experimental datasets of the XJTU-SY bearing from the group of Professor Yaguo Lei [31] and the early fault stage of bearing experiment data from the group of Professor Huaqing Wang [32] were used for analysis, and the IACMDSR results are compared with a standard MCKDSR model (MCKD joint sparse representation) and a leading MCKDMWF (MCKD joint Morlet wavelet filter).

### 5.1. XJTU-SY Bearings Compound Fault Data

**Figure 11:**
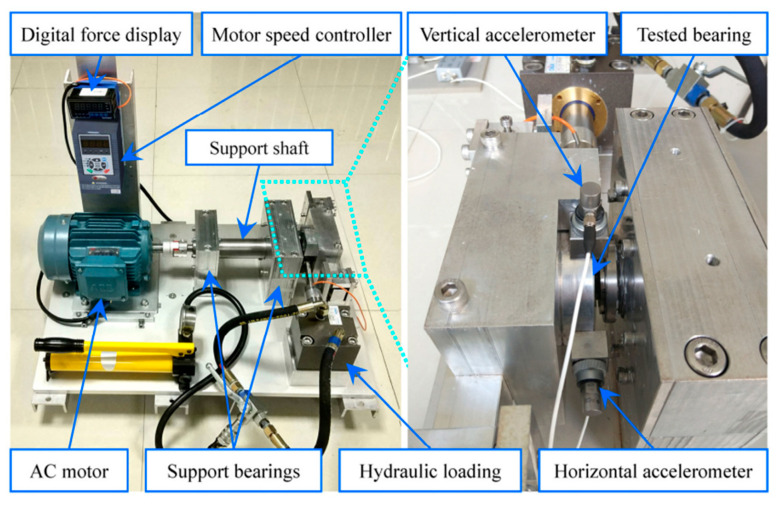
The tested bench.

**Figure 12:**
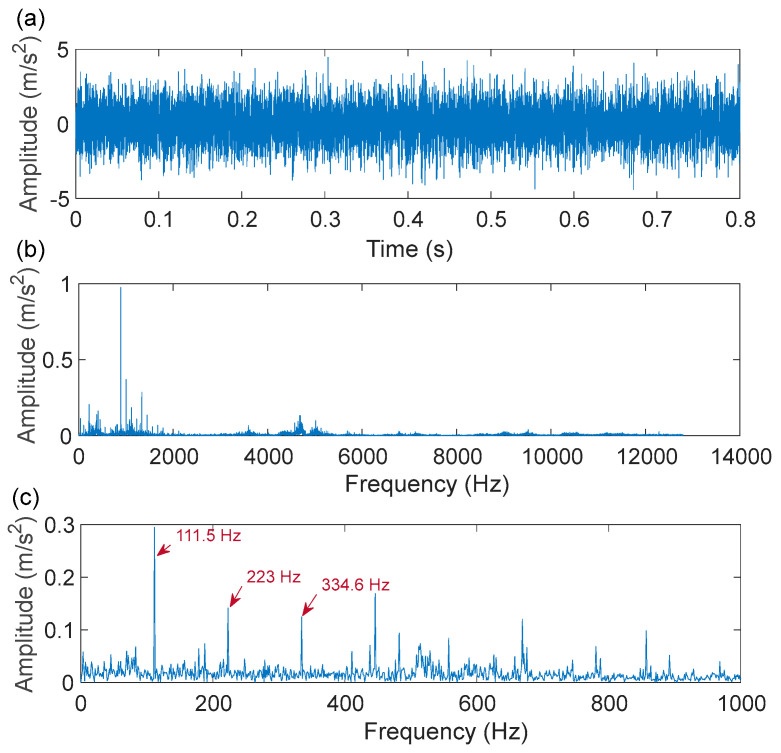
Compound fault measurement signal of the XJTU-SY bearing: (**a**) time-domain waveform; (**b**) spectrum; (**c**) envelope spectrum.

As shown in Figure 11, a motor speed controller, a hydraulic loading system, an alternating current (AC) induction motor, and a support shaft make up the run-to-failure test bench. Two acceleration sensors are placed in the bearing housing, one positioned vertically and the other horizontally. The type of bearing is LDK UER204, which is an external spherical bearing with concentric sleeve locking. The signal sampling frequency was set at 25.6 kHz. Table 1 displays the detailed parameters and the fault frequency of the bearing. At the rotating speed of 900 rpm, the bearing compound fault of the inner ring and outer ring is analyzed. According to the formula of the bearing fault characteristic frequencies, the fault characteristic frequency of the inner ring and outer ring is 172.9 Hz and 107.9 Hz, respectively. Figure 12 depicts the time-domain waveform, spectrum, and envelope spectrum of the compound fault vibration signals. From the time-domain waveform presented in Figure 12a, we can see that the fault impulse responses in the compound signal are submerged in harmonic interference and noise, as indicated by its envelope spectrum presented in Figure 12c, where the right characteristic frequency of the inner ring fault (172.9 Hz) and outer ring fault (107.9 Hz) are not presented.

**Figure 13:**
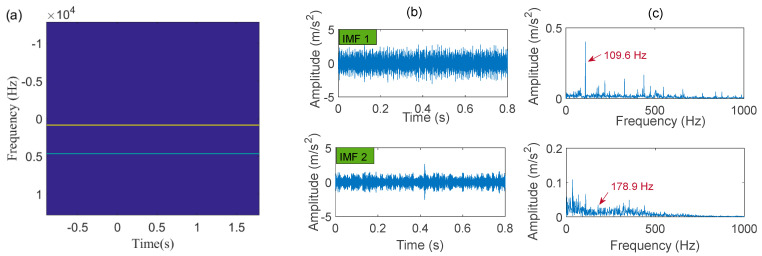
The processing results of the IACMD: (**a**) time-frequency graph; (**b**) the time-domain waveform; (**c**) the envelope spectrum.

**Figure 14:**
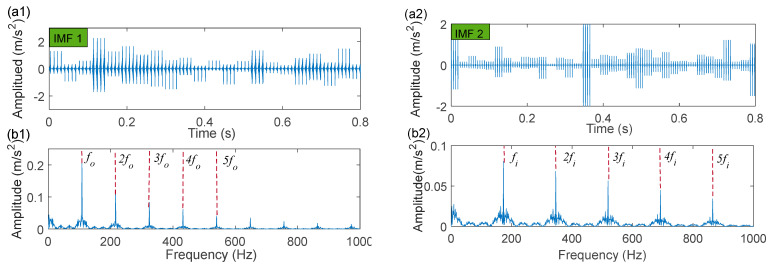
The IACMDSR model results of IMF1 (outer ring fault) and IMF 2 (inner ring fault): (**a1**,**a2**) time-domain waveform; (**b1**,**b2**) envelope spectrum.

**Figure 15:**
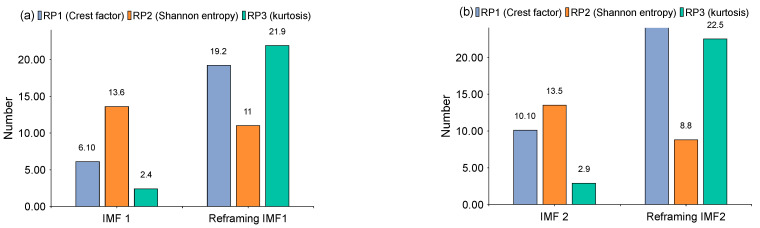
The index results of IACMD decomposition (Figure 13) and IACMDSR reconstruction (Figure 14) (**a**) IMF 1; (**b**) IMF 2.

Next, the IACMD is applied to isolate the compound signal (Figure 12a). Figure 13 displays the results of the IACMD. As shown in Figure 13a, two distinct fault resonance frequency bands are shown in the time-frequency graph. Figure 13b displays the time-domain waveform of the in-band signal components that are shown in Figure 13a. Nevertheless, these preliminary results are insufficient to accurately extract the fault feature. Therefore, the envelope spectrum (Figure 13c) shows frequencies with characteristics similar to the fault but with significant noise and interference components. In fact, the IACMD, as an adaptive filter, can distinguish the resonance frequencies of the different signal components, but the decomposed in-band components still contain a large amount of noise that cannot be removed. Therefore, this paper performs a sparse representation of the in-band fault signal to pick up the fault feature. As illustrated in Figure 14(a1,a2), the repetitive transients caused by the faults are clearly exhibited in the time-domain waveform, and the corresponding optimal parameters of the bilateral wavelet are: $f_{1}=874\;\mathrm{Hz},\;\xi_{1}=0.1999,\;\zeta_{1}=0.0513;f_{2}=4636$ Hz, $\xi_{2}=0.2,\;\zeta_{2}=0.0233$. As a result, the envelope spectrum is presented in Figure 14(b1,b2), where the fault characteristic frequency is effectively extracted. Likewise, Figure 15 depicts the quality results of the IACMD decomposed and further reconstructed signal components using the IACMDSR model. Therefore, we can conclude that IACMDSR has good performance in extracting fault features and diagnosing compound faults.

#### Comparison and Analysis of Experimental Results

(1) The MCKDSR Model

**Figure 16:**
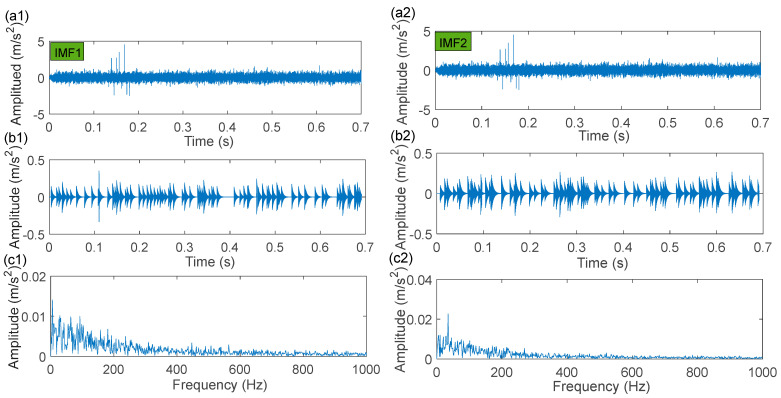
The MCKDSR processing results of IMF1 (outer ring fault) and IMF2 (inner ring fault): (**a1**,**a2**) filtered signal by the MCKD algorithm; (**b1**,**b2**) re-constructed signal; (**c1**,**c2**) envelope spectrum.

To demonstrate the superiority of the IACMD with sparse representation in this paper, the MCKD algorithm was used as an alternative to the IACMD to process the raw signal (Figure 12a). The MCKD algorithm has gained enormous application for separating compound faults [33,34]. By setting the parameter of the deconvolution period *T*, the deconvolution signal of interested component is extracted to realize the fault feature separation. The Laplace wavelet is in a shape similar to that of bearing fault-induced impulse responses with signal-sided attenuation [35]. Thus, in the comparison analysis, the Laplace wavelet parameter dictionary is used in a sparse representation. Here, the deconvolution period *T* of the MCKD algorithm is $T_{1}=235,\;T_{2}=148.$ $(T=f_{s}/f_{t})\;(f_{s}$ is the sampling frequency and $f_{t}$ is the corresponding fault frequency) [36]. The filter order is $L_{1}=L_{2}=$550 and the shift number is $M_{1}=M_{2}=$7. The selected parameters via the CFA method of the Laplace wavelet model for dictionary atom are as follows: $f_{1}=12,400,\;f_{2}=5100;\;\xi_{1}=0.015,\;\xi_{2}=0.0310$. Figure 16 shows the MCKDSR model processing results for the outer and inner ring fault signals, respectively. We can see from the time-domain waveforms (Figure 16(a1,a2)) after MCKD filtering that the main bearing fault features are significantly enhanced. Next, the time-domain waveforms of Figure 16(a1,a2) after further sparse representation are plotted in Figure 16(b1,b2), respectively. In each of them, certain fault impulses occurred, but the positions and periods of the impulses are inconsistent with the theoretical periodic impulses. Thus, in the envelope spectrum shown in Figure 16(c1,c2), no significant frequency of fault characteristic is presented.

(2) The MCKDMWF Model

**Figure 17:**
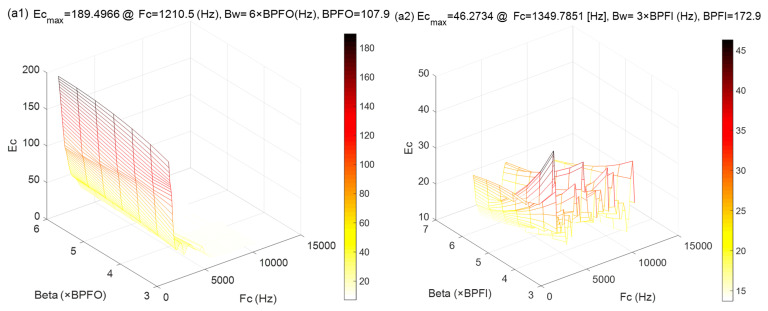

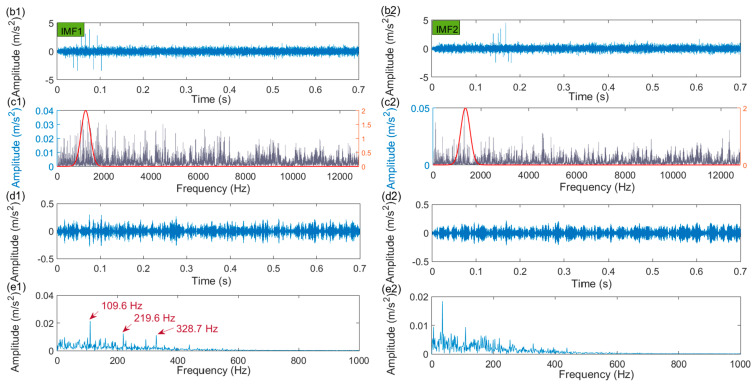
The MCKDMWT processing results of IMF1 (outer ring fault) and IMF2 (inner ring fault): (**a1**,**a2**) the parameter selection of the Morlet wavelet filter; (**b1**,**b2**) the filtered signal by the MCKD algorithm; (**c1**,**c2**) the shape of the Morlet wavelet filter and the spectrum; (**d1**,**d2**) filtered signal of the Morlet wavelet filter; (**e1**,**e2**) envelope spectrum.

The wavelet filter is a versatile tool that can efficiently extract potential fault features [37,38]. Therefore, in this comparison experiment, the MCKD algorithm is also applied to initially separate the different fault components, followed by the Morlet wavelet filter to extract the fault feature of the in-band signals. The parameters of the MCKD algorithm were set as in the MCKDSR model. For the Morlet wavelet filter, the filtering effect is determined by the bandwidth β and the central frequency $f_{c}$. The bandwidth is usually set to 3–4 times the maximum characteristic frequency. In order to exclude interference components and include bearing fault components as far as possible, the bandwidth is set to β=[3×BPFI,7×BPFI]. Next, with a defined bandwidth, the central frequency is derived in combination with the wavelet admissibility condition and the sampling theorem is: $\max(2.5\beta_{min},30f_{r}+\beta_{min}/2)<f_{c}<\min(f_{s}/2-\beta_{min}/2,0.8\times f_{s}/2)$. The crest factor of the envelope spectrum, which considers the amplitude and periodicity of the fault impulses, is chosen as an index for the Morlet wavelet filter parameters selection. Figure 17(b1,b2) illustrates the time-domain waveforms of the outer and inner ring fault signals isolated from the raw signal (Figure 12a) using the MCKD algorithm. The results of parameter selection for the corresponding Morlet wavelet are shown in Figure 17(a1,a2). Figure 17(c1,c2) shows the spectrum of Figure 17(b1,b2), as well as the shape of the optimal Morlet wavelet filter. Figure 17(d1,d2) illustrates the time domain signal after the Morlet wavelet filter. As illustrated in Figure 17(d1,d2), the fault impulse responses have improved markedly. Figure 17(e1,e2) shows the envelope spectrum for Figure 17(d1,d2). Despite the fact that the spectral peaks of IMF1 (outer race fault) can be seen in the envelope spectrum depicted in Figure 17(e1), they are still affected by noise to some extent. Worse, the no fault characteristic frequency of IMF2 (inner race fault) is visible in the envelope spectrum (Figure 17(e2)). As a result, we may conclude that the MCKDMWF model’s fault feature extraction effect is considerably inferior to that of the IACMDSR model.

### 5.2. The Bearing Early Fault Experiment Data

**Figure 18:**
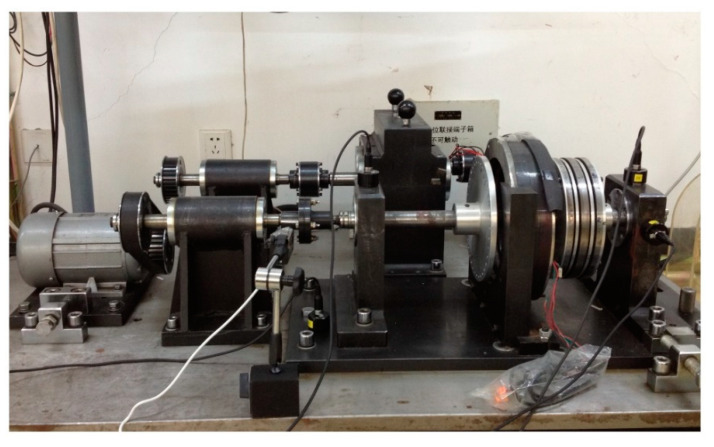
The experimental system for bearing fault diagnosis.

In the early stage of a bearing failure, the fault-induced impulse responses are normally very weak and are accompanied by strong confusion noise. Therefore, the feature extraction and identification of early compound faults are even more challenging. In this section, the efficiency of the proposed approach in early compound fault of bearings is validated. Figure 18 illustrates the installation positions of the acceleration sensor in the experiment system. The compound fault of the bearing outer-race, the unbalance fault, and the roller fault is the analysis goal. A groove with a width of 0.5 mm and a depth of 0.15 mm was machined into the outer ring and the roller of the bearing, respectively. A sampling frequency of 100 kHz and the rotating speed of 900 rpm were adopted in this experiment. The type of the fault bearing is NTN N204, and the detailed parameters of the bearing are given in Table 2. According to the theoretical formulae of fault characteristic frequency, the characteristic frequency for outer ring fault and rollers are 59.8 Hz and 71.8 Hz, respectively.

**Figure 19:**
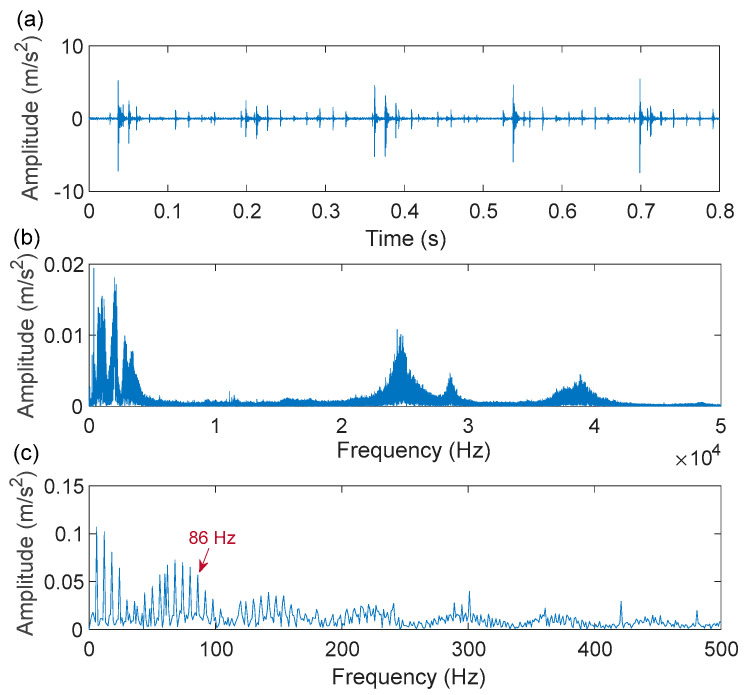
The early compound fault signal: (**a**) time-domain waveform; (**b**) spectrum; (**c**) envelope spectrum.

**Figure 20:**
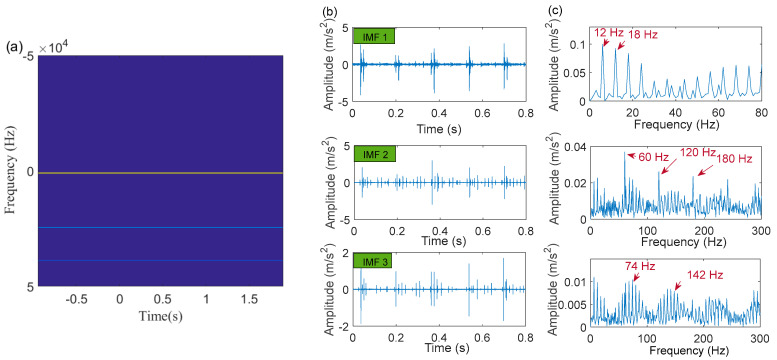
The processing results by IACMD: (**a**) time-frequency graph; (**b**) time-domain waveforms; (**c**) envelope spectrum.

The fault impulse responses of the compound fault signal are irregular, as shown in the time-domain waveform plotted in Figure 19a, and as illustrated by its envelope spectrum given in Figure 19c, where no significant frequency of the fault characteristics is presented. Next, the IACMD is employed for the pre-processing of the compound signal (Figure 19a). Figure 20a shows the multiple resonance frequencies extracted by the IACMD. In addition to the resonance frequencies formed by the outer ring and roller faults, the resonance band caused by the bearing unbalance fault is also clearly identified. Figure 20b,c displays the time-domain waveforms and the envelope spectrums of the three components separated by the IACMD. Although spectral peaks and their multiplier appear in the envelope spectrum, they are not consistent with the right fault characteristic frequencies. This indicates that the impulse responses generated by the various faults in the original signal (Figure 19a) are not fully extracted by the IACMD because of the influence of noise as well as interference components.

**Figure 21:**
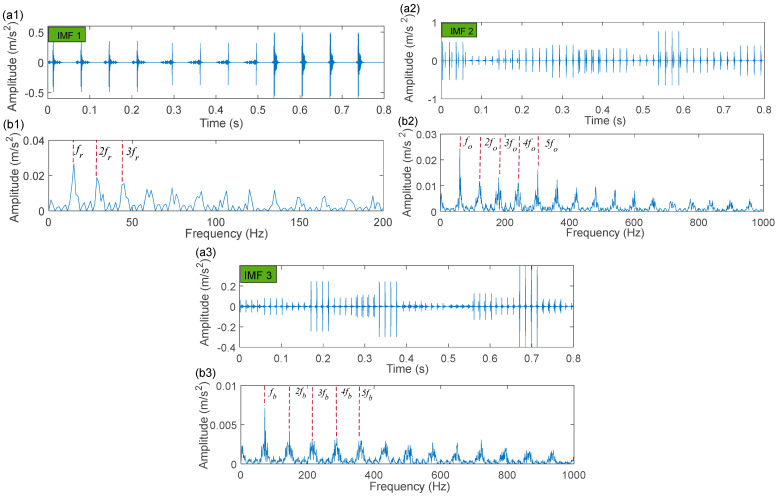
The IACMDSR model results of IMF1 (unbalance fault), IMF2 (outer ring fault), and IMF3 (roller fault): (**a1**–**a3**) time-domain waveform; (**b1**–**b3**) envelope spectrum.

The IACMD-decomposed components are then sparsely characterized using the sparse representation method outlined in Section 3. The final results of IACMDSR model for each component (IMF1 (unbalance fault), IMF2 (outer race fault), and IMF3 (roller fault)) are shown in Figure 21. The corresponding parameters are: $f_{1}=931\;\mathrm{Hz},\;\xi_{1}=0.1719,\;\zeta_{1}=0.0530;\;f_{2}=2459\;$ Hz, $\xi_{2}=0.1719,\;\zeta_{2}=0.0233$;$\;f_{3}=3874\;\mathrm{Hz},\;\xi_{3}=0.0014,\;\zeta_{3}=0.0029$. The time-domain waveforms of the reconstructed signals by the IACMDSR model are exhibited in Figure 21(a1–a3), which clearly shows that almost all of the repetitive impulse responses induced by the fault are captured. The spectrum peaks indicative of the corresponding fault characteristic frequencies and their harmonics are prominent in Figure 21(b1–b3). As a result, we can conclude that the primary fault features of the outer ring, the unbalance, and the rollers have been extracted essentially.

#### Comparison and Analysis

(1) The MCKDSR Model

**Figure 22:**
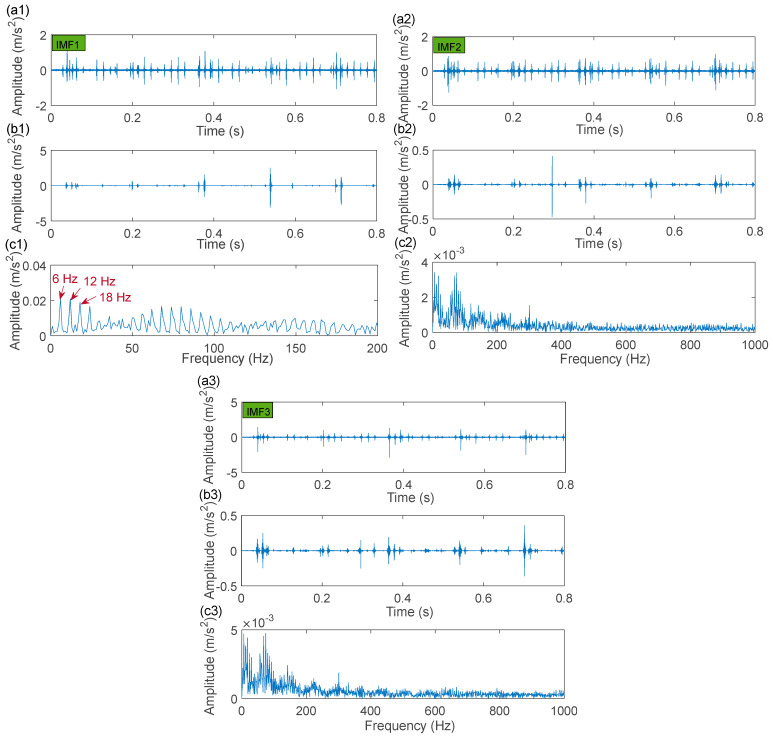
The MCKDSR processing results of IMF1 (unbalance fault), IMF2 (outer ring fault), and IMF3 (roller fault): (**a1**–**a3**) filtered signal by MCKD algorithm; (**b1**–**b3**) re-constructed signal; (**c1**–**c3**) envelope spectrum.

Similarly, the MCKDSR model was adapted to handle the identical vibration signal (Figure 19a) in order to demonstrate the superiority of the proposed technique. In this experiment data, the deconvolution period *T* of the MCKD algorithm is $T_{1}=6666,\;T_{2}=1672,\;T_{3}=1392$. The filter order and the shift number are also $L_{1}=L_{2}=L_{3}=$ 550, and $M_{1}=M_{2}=M_{3}=$ 7, respectively. The corresponding parameters of the Laplace wavelet as the dictionary atom by the CFA are $f_{1}=900,\;f_{2}=3600,f_{3}=3900;\;\xi_{1}=0.001,\;\xi_{2}=0.001,\;\xi_{3}=0.001$. The processing results of the MCKDSR model for the three signal components are exhibited in Figure 22. It can be calculated that a bearing with a rotational frequency of 900 rpm would have an unbalance fault frequency of 15 Hz. However, the correct characteristic frequency does not appear in the envelope spectrum of the unbalance fault signal component (IMF1) in Figure 22(c1). The MCKDSR model also failed to capture significant fault features from the processing results of the outer ring fault signal component (IMF2) and the roller signal component (IMF3). As a result, no fault characteristic frequencies are presented in the corresponding envelope spectrum. Actually, the oscillation frequencies extracted by the CFA do not match the energy concentration part of the original signal spectrum plotted in Figure 19b, whereas the resonance frequencies extracted by the IACMD are in general agreement with the range containing the three energy peaks of Figure 19b.

(2) The MCKDMWF Model

**Figure 23:**
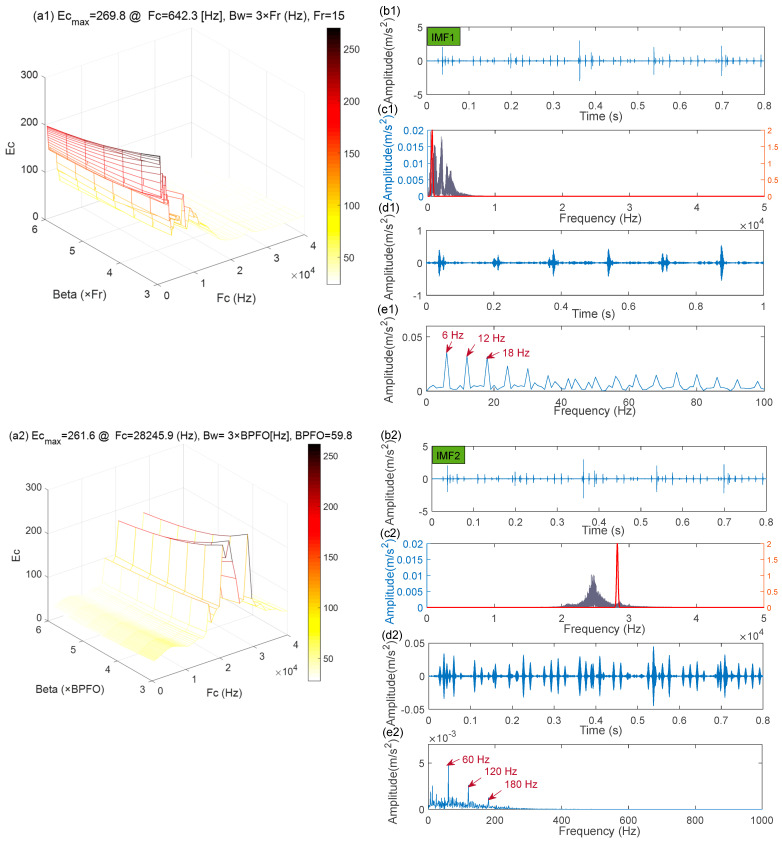

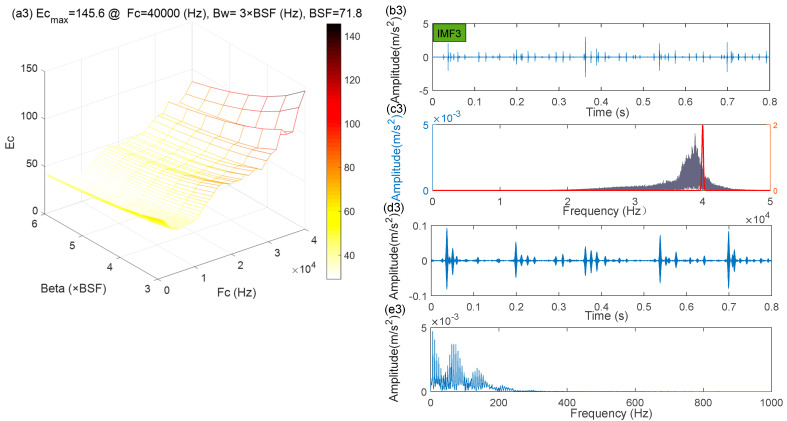
The MCKDMWT processing results of IMF 1 (unbalance fault), IMF 2 (outer race fault), and IMF 3 (roller fault): (**a1**–**a3**) the parameter selection of the Morlet wavelet filter; (**b1**–**b3**) filtered signal by MCKD algorithm; (**c1**–**c3**) the shape of the Morlet wavelet filter and the spectrum; (**d1**–**d3**) filtered signal of the Morlet wavelet filter; (**e1**–**e3**) envelope spectrum.

The MCKDMWF model is also used to handle the raw signal (Figure 19a). Finally, the analysis results of the three signal components by MCKDMWF are presented in Figure 23. Among the three sets of processing results, Figure 23(a1–a3) represents the parameter selection results of the Morlet wavelet filter, Figure 23(b1–b3) is the filtering result by the MCKD algorithm, Figure 23(c1–c3) gives the window of the Morlet wavelet filter and the spectrum of the Figure 23(b1–b3), Figure 23(d1–d3) is the filtered signal by the Morlet wavelet filter. As shown in the envelope spectrum (Figure 23(e1)), the MCKDMWT model was unable to extract the correct unbalance fault (IMF1) features, resulting in theoretical fault feature frequencies (15 Hz) not appearing in the corresponding envelope spectrum. In Figure 23(e2), a few noticeable spectral peaks of the outer race fault (IMF2) characteristic frequency may be observed but the result is significantly inferior to Figure 21(b2). Worse still, no useful fault feature was extracted from the rollers’ fault (IMF3) signal components, resulting in no significant roller fault characteristic frequencies emerging in Figure 23(e3). The aforementioned experiment results show that the IACMDSR model significantly outperformed the other modes in terms of multiple fault feature extraction, which provides an appealing tool for bearing compound fault detection.

## 6. Conclusions

In this paper, the IACMDSR model is put forward for vibration signal denoising and detecting the multiple fault signatures of the bearing. The IACMDSR model can not only automatically extract the multiple resonance frequencies and separate the signal components of a compound fault but can accurately recover the fault-induced impulse response of various signal components. The main conclusions are summarized as follows:

(1) The effectiveness of the IACMDSR model that incorporates IACMD with sparse representation is validated by simulation signals and two sets of experimental signals, and it exhibits better fault extraction performance than the MCKDSR model and MCKDMWF model. These experiment analyses confirm that the IACMDSR model is powerful and has the capability of detecting bearing weak multiple fault features in the presence of heavy noise, which is the Achilles’ heel of the two other leading rival methods.
(2) The performance of the IACMD in extracting fault resonance band is evaluated by the comparison with STFT, CWT, and ACMD, and the comparison results demonstrate that the IACMD exhibits better noise immunity. Additionally, the hyper-dictionary is developed by period-assisted bilateral wavelets, which simultaneously dig deeper into the periodicity and waveform characteristics exhibited by the real fault impulse response. Therefore, it is more suitable for practical engineering applications.
(3) The developed IACMDSR model can be extended to those diagnosis fields such as railway axles, high-speed train gearboxes, as well as engine diagnosis. It is worth pointing out that the IACMDSR has outstanding anti-noise capabilities and self-adaptability. Thus, it is completely suitable for the fault feature extraction of railway bearings with heavy noise and a complex working environment, which can play a significant role in maintaining the safety and comfort of the railway transport system.

Further research will focus on adjusting for the variable amplitude of the reconstructed signal. We would like to embed the feature enhancement algorithm into the sparse representation model. In addition, the characteristics of bearing fault vibration signals under variable speed conditions will be investigated in order to build a sparse representation compound dictionary suitable for vibration signals under variable conditions.

## Figures and Tables

**Table 1:** The parameters of LDK UER204 bearing.

Geometric Parameter	Numerical
Rolling diameter *Dr* (mm)	7.92
Inner ring raceway diameter *Di* (mm)	29.30
Outer race diameter *Do* (mm)	39.8
Bearing mean diameter (mm)	34.55
Contact angle (α/°)	0
Rollers number *Z*	8
Load rating (static) (kN)	6.65
Load rating (dynamic) (kN)	12.82

**Table 2:** The parameters of NTN N204 bearing.

Geometric Parameter	Numerical
Inner race diameter *Di* (mm)	20
Outer race diameter *Do* (mm)	47
Contact angle (α/°)	0
Rollers number *Z*	10
Thickness (mm)	14

## Data Availability

The data used to support the finding of this study are available from the corresponding author upon request.
